# Genetic variability in LMP2 and LMP7 is associated with the risk of esophageal squamous cell carcinoma in the Kazakh population but is not associated with HPV infection

**DOI:** 10.1371/journal.pone.0186319

**Published:** 2017-10-26

**Authors:** Lan Yang, Yu Ji, Ling Chen, Mei Li, Fei Wu, Jianming Hu, Jinfang Jiang, Xiaobin Cui, Yunzhao Chen, Lijuan Pang, Yutao Wei, Feng Li

**Affiliations:** 1 Department of Pathology and Key Laboratory for Xinjiang Endemic and Ethnic Diseases, Shihezi University School of Medicine, Shihezi, Xinjiang, China; 2 The First Affiliated Hospital, Shihezi University School of Medicine, Shihezi, Xinjiang, China; 3 Department of Pathology, Beijing ChaoYang Hospital, Capital Medical University, Beijing, China; Fondazione IRCCS Istituto Nazionale dei Tumori, ITALY

## Abstract

The Kazakh population in Xinjiang Province in northwestern China exhibits a high incidence of esophageal squamous cell carcinoma (ESCC). Although the etiology of esophageal carcinoma (EC) has not been elucidated, there are reports of the involvement of an immunologic mechanism. In the current study, 268 Kazakh ESCC patients and 500 age- and sex-matched control subjects were recruited. DNA was extracted from paraffin-embedded tumor specimens from the patients and peripheral blood lymphocytes from the controls and used for LMP2/LMP7 genotyping. Polymerase chain reaction-restriction fragment length polymorphism (PCR-RFLP) analysis was performed to detect LMP2/LMP7 gene single-nucleotide polymorphisms (SNPs). We found a clear increased risk of ESCC in the Kazakh population for the heterozygous LMP2 R/C genotype and the homozygous C/C genotype (OR = 1.470, 95%CI = 1.076–2.008, *p* = 0.015 forLMP2R/C; OR = 2.048, 95% CI = 1.168–3.591, *p* = 0.011 for LMP2 C/C). Conversely, the heterozygous LMP7 Q/K polymorphism was found to decrease the risk of ESCC in this population (OR = 0.421, 95% CI = 0.286–0.621, *p* = 8.83×10^−6^). Moreover, LMP2 R/C+C/C genotype was associated with increased tumor invasion depth (*p* = 0.041). Haplotype analysis showed that haplotype A, which includes wild-type homozygous LMP2/TAP1 and mutant LMP7, decreases susceptibility to ESCC in the Kazakh population; in contrast, haplotype E, which includes wild-type homozygous LMP2/LMP7/TAP1, acts as a risk factor for increased susceptibility to ESCC. This is the first study to report that the heterozygous LMP2 R/C and homozygous C/C genotypes increase susceptibility to ESCC in the Kazakh population and that the heterozygous LMP7 Q/K genotype decreases susceptibility to ESCC in this population. Nevertheless, neither LMP2 nor LMP7 was associated with human papillomavirus (HPV) infection. Understanding LMP2/LMP7 genetic variability will provide a new therapeutic perspective for Kazakh patients with ESCC.

## Introduction

Esophageal carcinoma (EC) is considered one of the most malignant cancers worldwide [1–5]. More than 90% of ECs are either esophageal squamous cell carcinomas (ESCCs) or adenocarcinomas (EACs). It is reported that EAC is markedly more prevalent in western countries; in contrast, EC cases are almost entirely ESCC in eastern and African countries [2, 6, 7]. Some etiologic and risk factors contribute to this phenomenon. In western countries and areas with a low incidence of ESCC, excess alcohol intake in combination with smoking greatly increases the risk of EC [8]. In developing countries, diets deficient in vitamins as well as intake of carcinogens have been speculated as risk factors for ESCC [9]. The Kazakh population, a minority group in the Xinjiang region of northwest China that still maintains its traditional way of life (such as living in a dry, hot climate and consuming a vitamin-deficient diet), exhibits the highest ESCC mortality rate among different minorities in China. Indeed, the death rate in this population can be up to 68.95/100,000 [10]. Therefore, it is urgent to identify ways to reduce the mortality rate in this population. Recently, some researchers showed that endogenous factors, such as the immune system and genetic factors, may be related to ESCC. Moreover, many genetic variations, especially polymorphisms, are prevalent in the Kazakh population, some of which are associated with an increased risk of cancer [11].

The low-molecular-weight polypeptide (LMP) system recognizes intracellular infection and plays a crucial role in immunological surveillance in humans through the MHC-I molecule and cytotoxic T-lymphocyte (CTL) pathway [12, 13]. LMP genes are located in the MHC class II region of chromosome 6p21.3 [14]. The genes LMP2 and LMP7 encode two proteasome subunits that are considered to function in the degradation of cytosolic proteins and the production of antigenic peptides [15]. After antigenic peptides are degraded by LMP2 and LMP7, they are transferred into the endoplasmic reticulum (ER) lumen by the transporter associated with antigen processing (TAP) protein, loaded onto MHC class I molecules, and presented to cytotoxic T lymphocytes [16]. Thus, because LMPs participates in antigen presentation, they play vital roles in autoimmune diseases and tumorigenesis [17–21]. A series of previous studies found that polymorphisms in LMP2 codon 60 (LMP2–60) (Arg–Cys) and LMP7 codon 145 (LMP7–145) (Gln–Lys) are responsible for functional evolution and thus reduce the ability of these molecules to function in antigen processing [22, 23]. Accordingly, these genetic variants in LMP2/LMP7 have been linked to the occurrence, development, and prognosis of many diseases, including viral infection, autoimmune disease, and malignant tumors [17–20].

Many studies to date have found that LMP2/LMP7 polymorphism affects susceptibility to numerous cancers. For instance, the LMP7–145 gene polymorphism contributes to increased susceptibility to gastric cancer and ovarian cancer [11, 24]. LMP7–145 lysine carriers in the Han population in Anyang, Henan, China, were also found to be more susceptible to EC [22]. In contrast, no statistical correlation has yet been found between LMP2–60 polymorphic genotypes and the risk of ovarian or lung cancers [11, 25]. However, the relationship between LMP2/LMP7 polymorphism and the risk of ESCC in the Kazakh population remains unclear. Therefore, studying LMP2/LMP7 genetic variability in Kazakh patients with ESCC is important for providing a new therapeutic perspective.

## Materials and methods

### Specimens

Two hundred sixty-eight formalin-fixed and paraffin-embedded ESCC tissues were obtained from 268 Kazakh patients who had been treated with esophagectomy without any additional therapy, such as radiation and chemotherapy, during the period from January 1990 to March 2014 at People’s Hospital of Xinjiang Uygur Autonomous Region, First Affiliated Hospital of Shihezi University Medical College in Xinjiang and some hospitals in the Yili Kazakh Autonomous Region. All 268 of the cases originated from the ESCC-high-risk Kazakh population in Xinjiang. The age of the study group ranged from 23 years to 79 years, with a mean of 53.14±8.20 years. A standardized questionnaire was used to collect information on demographic characteristics (gender, age, area of birth and residence) and life-style variables, including consumption of hot tea, alcohol and tobacco(S3 Table),we defined drinkers as drinking more than 20g per day,greater than 5 times a week,drinking time is greater than 10 years. Smokers are defined as smoking more than 1 cigarettes one day, smoking time is more than 6 months. In order to ensure the reliability of the results of the survey and the data collected by the authenticity, we carried out the analysis of reliability and validity of the questionnaire before the formal investigation, and according to the analysis results, then screened second issues, adjusted the overall structure of the questionnaire.Pathological diagnoses of tumor-node-metastasis (TNM) stages were estimated for all cases using guidelines provided in Cancer Stage Manual 7th Edition (2009) issued by the American Joint Committee on Cancer/Union for International Cancer Control (AJCC/UICC). For controls, we randomly selected 500 Kazakh volunteers from the same geographic area who had no cancer history and were gender- and age-matched with the cases. Blood samples from these 500 controls were collected, and DNA was extracted for LMP gene genotyping. All participants in the study signed an informed consent form. First Affiliated Hospital of Shihezi University School of Medicine Institutional Ethics Review Board approved this research. We conducted the experiment in 2015.

### Genotyping of LMP2/ LMP7 polymorphisms and haplotype construction

DNA was extracted from paraffin sections and blood samples using standard proteinase K digestion and a tissue DNA extraction kit following the manufacturer’s instructions (QIAGEN, Hilden, Germany). The two previously documented polymorphisms in the LMP2/LMP7 coding regions are listed in S1 Table. The polymerase chain reaction (PCR) primers used to assess the polymorphisms mentioned above are listed in S1 Table. PCR was performed in a total volume of 25 μl containing 1 μl DNA, 2.5 μl 10× PCR buffer (100 mM Tris, pH 7.8, 100 mM NaCl, 10 mM ethylenediaminetetraacetic acid (EDTA) and 0.5% sodium dodecyl sulfate (SDS)), 1.8 μl 25 mM MgCl_2_, 0.5 ml each dNTP, 10 pmol primers and 1.5 U Taq Polymerase (Promega, Madison, WI, USA). For PCR amplification, the reaction was initially maintained at 94°C for 2 min, followed by 30 cycles of the following conditions: denaturation at 94°C for 40 s, annealing at 57.5°C for 40 s, elongation at 72°C for 40 s. A final extension at 72°C was performed for 10 min. Gene-Amp PCR System 9700 was used. The PCR products were purified using a MultiScreen-PCR purifying plate (Millipore Company, USA).

For restriction fragment length polymorphism (RFLP) analysis, 330-bp and 351-bp PCR products for the LMP2–60 and LMP7–145 polymorphisms were digested using the specific restriction endonucleases HhaI and BsmI (New England Biolabs, Ipswich, MA, USA). The PCR products were digested under the conditions recommended by the manufacturer and then electrophoresed through a 3% agarose gel.

The wild-type genotype of the LMP2–60 polymorphism yielded bands of 79 and 251 bp, and the heterozygous genotype yielded bands of 79, 251 and 330 bp (S1 Fig). Regarding LMP7–145, bands of 146 and 205 bp for the Gln/Gln genotype, a band of 351 bp for the Lys/Lys genotype, and bands of 146, 205 and 351 bp for the Gln/Lys genotype were obtained (S2 Fig). Fragment sizes were evaluated by comparison with known markers (Fermentas, Inc, Glen Burnie, MD). DNA direct sequencing was used to confirm the genotypes of LMP2–60 and LMP7–145 (S1 Fig and S2 Fig), and the two results were 100% concordant. LMP2/LMP7 and TAP1 gene polymorphism haplotypes were constructed using PHASE [26] software.

### Detection of HPV DNA

DNA was extracted from 268 paraffin-embedded tissue samples from Kazakh patients using standard proteinase K digestion and a tissue DNA extraction kit (QIAGEN, Hilden, Germany). We used a human β-actin primer set as an internal control for PCR: forward, 5’-CAGACACCATGGTGCACCTGAC-3’, and reverse, 5’-CCAATAGGCAGAGAGAGTCAGTG-3’. We used non-degenerate primers to determine HPV infection (GP 5+/6+, forward primer 5’-TTGGATCCTTTGTACTGTGGTAGATACTAC-3’, and reverse primer 5’-TTGGATCCGAAAAATAAACTGTAAATCATATTC-3’), which amplify a 150-bp fragment of the L1 gene in a large range of HPV types. For HPV16 detection, E7 was amplified with forward primer 5’-GATGAAATAGATGGTCCAGC-3’ and reverse primer 5’-GCTTTGTACGCACAACCGAGC-3’. For each PCR reaction, the final volume was 25 μl and included 5 μl extracted concentrated DNA. The reaction was first carried out at 95°C for 10 min, followed by 40 cycles at 94°C for 30 s, 42°C for 90 s, 72°C for 30 s, and a final extension at 72°C for 5 min. The amplicons were then denatured and subjected to hybridization. The samples were tested in triplicate, and β-actin was used as an internal control.

### Statistical analysis

All statistical analyses were performed using SPSS version 17.0 (U.S.). Hardy-Weinberg equilibrium (HWE) for the cases and controls was evaluated using chi-square tests to compare the observed and expected genotype frequencies (S2 Table). Allele and genotype frequencies were compared by contingency table analysis using Fisher’s exact test if the number of observations in any cell was less than five. The *p* values were corrected based on the number of specimens tested and the number of comparisons performed. Student’s *t*-test and the χ^2^ test were utilized to calculate the significance of differences in continuous variables between LMP genotypes. The relationship between single-nucleotide polymorphisms (SNPs) in LMP2/LMP7 and the risk of ESCC was estimated by determining the odds ratios (ORs) at 95% confidence intervals (CIs) using logistic regression analyses for crude ORs and adjusted ORs when adjusting for age and sex. Logistic regression was also applied to analyze the HPV status and risk of ESCC.

## Results

### Characteristics of the study population

The characteristics of the 268 cases and 500 controls included in this study are summarized in Table 1. The age was 53.14 ± 8.20 (mean ± SD) years for the ESCC cases and 54.59±10.75 (mean ± SD) years for the controls. No significant difference was observed for age and gender (*p* = 0.095 and *p* = 0.639, respectively). Statistical analysis showed that smoking is closely linked to the risk of ESCC in the Kazakh population (*p* = 0.027), though drinking was not significantly associated (*p* = 0.393). We also evaluated data for the case group with regard to differentiation status, tumor location, depth of invasion and lymph node metastasis.

**Table 1:** Characteristics of ESCC cases and controls.

Variables	Cases[n(%)] n = 268	Controls[n(%)] n = 500	*p*^a^
Age(years) mean±SD	53.14±8.20	54.59±10.75	0.194
Age(years)			0.095
<57	172	290	
≥57	96	210	
Sex			0.639
Male	158	286	
Female	110	214	
Smoking			0.027*
Yes	119	264	
No	149	236	
Drinking			0.393
Yes	148	260	
No	120	240	
Differentiation status			
Well differentiated(G1)	81		
Moderately differentiated(2)	157		
Poorly differentiated(G3)	30		
Tumor location			
Upper	11		
Middle + lower	257		
Depth of invasion			
T1/T2	151		
T3/T4	117		
Lymph node metastasis			
No	151		
Yes	117		

^a^ Two-side x^2^ test and Student’s test

*means *p*<0.05

### Genotype frequencies of LMP2/LMP7 gene polymorphisms in cases and controls

The genotype frequencies of LMP2/LMP7 gene polymorphisms are presented in Table 2. In single-locus analyses of genotype frequencies, the frequencies for LMP2 genotypes were 41.4% (R/R), 48.5% (R/C), and 10.1% (C/C) among the cases and 52.2% (R/R), 41.6% (R/C), and 6.2% (C/C) among the controls. A significant difference was observed in this study. Relative to the R/R allele, R/C and C/C genotypes were associated with increased risk of ESCC in the Kazakh population (OR = 1.470, 95%CI = 1.076–2.008, *p* = 0.015 for R/C, OR = 2.048, 95%CI = 1.168–3.591, *p* = 0.011 for C/C, Table 2). The C allele also showed an increased risk of ESCC in the Kazakh population (OR = 1.413, 95% CI = 1.127–1.772, *p* = 0.003). In the dominant model, the RC+CC genotype was associated with a statistically increased risk of ESCC in the Kazakh population when the RR genotype was used as a reference group (RR versus RC+CC OR = 1.545, 95% CI = 1.145–2.085, *p* = 0.004, Table 3). However, in the recessive model, in which the RR+RC genotype was used as the reference group, the CC genotype was not associated with risk of ESCC in the Kazakh population (RR+RC versus CC OR = 1.695, 95% CI = 0.989–2.905, *p* = 0.053, Table 3).

**Table 2:** Genotype frequencies of LMP2/LMP7 gene polymorphisms in cases and controls.

genotype	case(n%) (n = 268)	control(n%) (n = 500)	*p*	OR(95%CI)	*p*^a^	Adjusted OR^a^ (95% CI)
LMP2						
R/R	111(41.4)	261(52.2)		1.000	1.000	
R/C	130(48.5)	208(41.6)	0.015*	1.470(1.076–2.008)	0.013	1.552(0.909–2.648)
C/C	27(10.1)	31(6.2)	0.011*	2.048(1.168–3.591)	0.011	1.086(0.382–3.086)
RR VS RC VS CC			0.008**		0.004*	
RC+CC	157	239	0.004**	1.545(1.145–2.085)	0.048	1.766(1.006–3.101)
RR+RC	241	469		1.000		
CC	27	31	0.053	1.695(0.989–2.905)	0.059	1.753(0.995–2.946)
R allele	352	730		1.000		
C allele	184	270	0.003**	1.413(1.127–1.772)		
LMP7						
Q/Q	218(81.3)	342(68.4)		1.000	1.000	
Q/K	40(14.9)	149(29.8)	<0.001**	0.421(0.286–0.621)	<0.001**	0.433(0.301–0.643)
K/K	10(3.73)	9(1.80)	0.229	1.743(0.697–4.358)	0.238	1.746(0.706–3.986)
QQ VS QK VS KK			<0.001**		<0.001**	
QK+KK	50	158	<0.001**	0.496(0.346–0.172)	<0.01**	0.902(0.377–1.774)
QQ+QK	258	391		1.000		
KK	10	9	0.259	1.684(0.675–4.201)	0.265	1.705(0.668–4.132)
Q allele	476	834		1.000		
K allele	60	166	0.004**	0.633(0.462–0.869)		

^a^Logistic regression adjusted for age,sex,smoking and drinking

“*”represents *p*<0.05

“**”indicates *p*<0.01

**Table 3:** Stratification analyses between LMP2 polymorphism and Kazakh ESCC patient clinicopathological parameters.

Parameter	RR	RC	CC	RR	RC		CC			RC+CC		RR+RC	CC	
					OR(95%CI)	P		OR(95%CI)	P	OR(95%CI)	P		OR(95%CI)	P
Gender ^a^														
Male	70/157	73/112	15/17	1.00	1.462(0.972–2.198)	0.067		1.979(0.935–4.186)	0.070	1.530(1.035–2.262)	0.033*	1.00	1.660(0.805–3.421)	0.166
Female	41/104	57/96	12/14	1.00	1.506(0.925–2.453)	0.099		2.174(0.928–5.095)	0.070	1.591(0.994–2.547)	0.052	1.00	1.749(0.780–3.924)	0.171
Age ^a^														
<57	73/137	83/135	16/18	1.00	1.154(0.778–1.711)	0.541		1.668(0.803–3.465)	0.234	1.214(0.830–1.776)	0.366	1.00	1.550(0.768–3.126)	0.295
≥57	38/124	47/73	11/13	1.00	2.101(1.254–3.520)	0.004*		2.761(1.144–6.666)	0.038	2.201(1.344–3.603)	0.002*	1.00	1.961(0.845–4.553)	0.173
Smoking^a^														
Yes	45/133	68/121	6/10	1.00	1.661(1.059–2.605)	0.027*		1.773(0.610–5.155)	0.288	1.670(1.073–2.598)	0.024*	1.00	1.349(0.479–3.801)	0.570
No	66/128	62/87	21/20	1.00	1.382(0.889–2.148)	0.150		2.036(1.031–4.022)	0.058	1.504(0.996–2.273)	0.066	1.00	1.764(0.920–3.379)	0.120
Drinking^a^														
Yes	53/120	83/125	12/15	1.00	1.503(0.982–2.302)	0.076		1.811(0.794–4.133)	0.154	1.536(1.-14-2.327)	0.054	1.00	1.441(0.656–3.168)	0.361
No	58/141	47/83	15/16	1.00	1.377(0.860–2.204)	0.182		2.279(1.057–4.912)	0.053	1.522(0.980–2.366)	0.061	1.00	2.000(0.953–4.198)	0.063
Tumor depth ^b^														
T1/T2	54	79	18	1.00	1.607(0.959–2.692)	0.072		2.112(0.863–5.167)	0.101	1.679(1.023–2.756)	0.041*	1.00	0.592(0.254–1.379)	0.224
T3/T4	57	51	9											
Histologic grade ^b^														
G1	36	36	9	1.00	0.806(0.462–1.405)	0.447		1.024(0.417–2.511)	0.959	0.847(0.499–1.437)	0.538	1.00	0.848(0.363–1.981)	0.704
G2/G3	75	94	18											
Lymph node metastasis ^b^														
Yes	42	64	11	1.00	0.619(0.368–1.041)	0.070		0.843(0.353–2.012)	0.701	0.659(0.400–1.084)	0.100	1.00	0.893(0.397–2.009)	0.784
No	69	66	16											
TNM stage ^b^														
I+II	83	95	20	1.00	0.895(0.500–1.601)	0.708		0.903(0.338–2.413)	0.839	0.906(0.518–1.583)	0.728	1.00	0.980(0.394–2.433)	0.964
III+IV	28	35	7											
HPV status ^b^														
HPV(+)	37	37	10	1.00	1.326(0.689–2.550)	0.398		0.825(0.280–2.430)	0.728	1.195(0.638–2.239)	0.577	1.00	1.497(0.538–4.167)	0.440
HPV(-)	32	45	7											

^a^ Stratification analysis to evaluate the effects of LMP2 genotypes on the risk of ESCC by age /sex/smoking/drinking.

^b^ Logistic regression analysis adjusted for age /sex/smoking/drinking.

“*”represents *p*<0.05

A significant difference was also observed for LMP7, with genotype frequencies of 81.3% (Q/Q), 14.9% (Q/K), and 3.73% (K/K) among the cases and 68.4% (Q/Q), 29.8% (Q/K), and 1.8% (K/K) among the controls. Compared with the Q/Q genotype, the Q/K genotype decreased the risk of ESCC in the Kazakh population (OR = 0.421, 95%CI = 0.286–0.621, *p*<0.001,Table 2). The K allele also decreased the risk of ESCC (OR = 0.633, 95% CI = 0.462–0.869, *p* = 0.004). The dominant model, for which the QQ genotype was used as a reference group, showed that the QK+KK genotype was associated with a statistically increased risk of ESCC in the Kazakh population (QQ versus QK+KK (OR = 0.496, 95%CI = 0.346–0.172, *p*<0.001), Table 3). In contrast, the KK genotype was not associated with the risk of ESCC in the Kazakh population in the recessive model (QQ+QK versus KK (OR = 1.684, 95% CI = 0.675–4.201, *p* = 0.259), Table 2). We used the expression of quantitative trait loci (eQTL) data which have been reported (https://gtexportal.org/home/) to study the effects of SNP locus of LMP2/LMP7 gene on gene expression, the results showed that SNP locus of LMP2 in esophageal tissues affected the expression of LMP2 (p = 1.30×10–7, S3 Fig). The SNP locus of LMP7 was not related to the expression of LMP7.This phenomenon may be due to a same sense mutation in the SNP locus of LMP7. Although the base substitution have been replaced, the protein levels have not been altered, and amino acids have not been replaced.

### Correlations between clinicopathological parameters and LMP2/LMP7 polymorphism in Kazakh patients with ESCC

To evaluate the contribution of confounding factors, including gender /age/ smoking and drinking to the risk of ESCC in the Kazakh population, stratification analyses were performed to investigate the potential effect of genetic variants of LMP2/LMP7 with ESCC risk in population subgroups (Table 3 and Table 4). This analysis showed that the LMP2–60 R/C and R/C+C/C genotypes were associated with an increased risk of ESCC in Kazakh patients aged ≥57 years but not in those aged <57 years. A significantly elevated risk was also identified in male subjects. In contrast, the LMP7–145 Q/K and QK+KK genotypes were associated with a decreased risk of ESCC in male patients aged ≥57 years but not in male patients aged <57 years. This analysis explained that the LMP2–60 R/C and R/C+C/C genotypes were associated with an increased risk of ESCC in Kazakh patients with smoking but not in those without smoking(*p*<0.05). There was no statistically significant with drinker and nondrinker(*p*>0.05). Equally contrary, the LMP7–145 Q/K and QK+KK genotypes were associated with a decreased risk of ESCC with smoking people but not in those without smoking(*p*<0.05). Nevertheless, the result indicated that LMP7–145 Q/K and QK+KK genotypes were associated with a decreased risk of ESCC who don't drink but not in those with drinking(*p*<0.05). When Kazakh ESCC patients were divided into two subgroups, T1/T2 and T3/T4, according to the AJCC TNM classification of EC, the RC+CC genotype was significantly associated with ESCC tumor depth (OR = 1.679, 95% CI = 1.023–2.756, *p* = 0.041). However, there was no significant association between LMP2/LMP7 polymorphism and ESCC in the Kazakh population with respect to clinical pathological parameters (*p*>0.05).

**Table 4:** Stratification analyses between LMP7 polymorphism and clinicopathological parameters of Kazakh ESCC patients.

Parameter	QQ		QK			KK	QQ	QK		KK		QK+KK		QQ+QK	KK	
								OR(95%CI)	P	OR(95%CI)	P	OR(95%CI)	P		OR(95%CI)	P
Gender ^a^																
Male	133/194		33/84			8/8	1.00	0.295(0.168–0.520)	0.000**	1.459(0.534–3.983)	0.459	0.396(0.242–0.650)	1.818×10^−4^**	1.00	1.853(0.682–5.037)	0.220
Female	85/148		23/65			2/1	1.00	0.616(0.350–1.063)	0.080	3.482(0.311–38.976)	0.282	0.660(0.387–1.123)	0.124	1.00	3.944(0.354–43.987)	0.229
Age ^a^																
<57	131/207		37/79			4/4	1.00	0.740(0.473–1.158)	0.187	1.580(0.388–6.428)	0.519	0.781(0.506–1.204)	0.262	1.00	1.702(0.420–6.896)	0.451
≥57	87/135		3/70			6/5	1.00	0.067(0.020–0.218)	0.000**	1.862(0.551–6.288)	0.310	0.186(0.089–0.391)	1.662×10^−6^**	1.00	2.733(0.813–9.188)	0.092
Smoking^a^																
Yes	95/152		20/109			4/3	1.00	0.294(0.171–0.504)	4.50×10^−6^**	2.133(0.467–9.742)	0.318	0.343(0.206–0.571)	2.529×10^−5^**	1.00	1.867(0.410–8.500)	0.413
No	123/190		20/40			6/6	1.00	0.772(0.431–1.383)	0.468	1.545(0.487–4.899)	0.457	0.873(0.513–1.486)	0.714	1.00	1.608(0.509–5.083)	0.414
Drinking^a^																
Yes	105/167		37/89			6/4	1.00	0.661(0.420–1.042)	0.094	2.386(0.658–8.654)	0.174	0.735(0.475–1.137)	0.203	1.00	2.704(0.751–9.742)	0.114
No	115/165		3/70			4/5	1.00	0.061(0.019–0.200)	0.000**	1.148(0.302–4.367)	0.840	0.155(0.068–0.349)	6.184×10^−7^**	1.00	1.729(0.456–6.555)	0.415
Tumor depth ^b^																
T1+T2	123		24			4	1.00	1.157(0.580–2.305)	0.679	0.501(1.136–1.842)	0.298	0.977(0.524–1.823)	0.943	1.00	2.021(0.552–7.391)	0.288
T3+T4	95		16			6										
Histologic grade ^b^																
G1	68		10			3	1.00	0.730(0.331–1.580)	0.424	0.941(0.236–3.756)	0.932	0.976(0.577–1.651)	0.927	1.00	1.029(0.259–4.094)	0.967
G2+G3	150		30			7										
Lymph node metastasis ^b^																
Yes	91		20			6	1.00	0.722(0.367–1.420)	0.345	0.481(0.13201.757)	0.268	0.670(0.361–1.243)	0.204	1.00	1.939(0.533–7.054)	0.315
No	127		20			4										
TNM stage ^b^																
I+II	162		30			6	1.00	1.041(0.478–2.268)	0.919	0.520(0.141–1.926)	0.328	0.893(0.448–1.779)	0.747	1.00	1.928(0.526–7.069)	0.322
III+IV	56		10			4										
HPV status ^b^																
HPV(+)	71		10			3	1.00	1.549(0.642–3.737)	0.330	1.101(0.211–5.750)	0.909	1.465(0.658–3.260)	0.350	1.00	0.932(0.178–4.879)	0.934
HPV(-)	66		15			3										

^a^ Stratification analysis to evaluate the effects of LMP7 genotypes on the risk of ESCC by age /sex/smoking/drinking.

^b^ Logistic regression analysis adjusted for age/gender/smoking/drinking.

“**”represents *p*<0.01

### Linkage disequilibrium and haplotype analysis of LMP2 /LMP7 and TAP1

The association between TAP1 polymorphisms and ESCC in the Kazakh population was shown in a previous study (40). Thus, haplotype analysis was performed to further evaluate the combined effects of LMP2/LMP7 and TAP1 polymorphisms on the risk of ESCC in this population. Among the eight haplotypes derived from the observed genotypes, A and E were the most common in both the cases and controls (A: 33.77% of patients and 45.5% of controls, E: 28.73% of patients and 17.6% of controls) (Table 5). The results showed that haplotype A, which includes wild-type homozygous LMP2/TAP1 and mutant LMP7, decreases susceptibility to ESCC in the Kazakh population (OR = 0.37, 95%CI = 0.23–0.59, *p*<0.001), whereas haplotype E, which includes wild-type homozygous LMP2/LMP7/TAP1, acts as a risk factor to increase susceptibility to ESCC in the Kazakh population (OR = 3.67, 95% CI = 1.85–7.28, *p*<0.001). The results are summarized in Table 6.

**Table 5:** Distribution of estimated haplotype frequencies for LMP/TAP genes in cases and controls.

Haplotype	Loci	Cases n (%)	Controls n (%)
Number of chromosome		536 (100)	1000(100)
Location	[LMP2]-[LMP7]-[TAP1-2]		
A	*Arg—Lys—Asp*	181 (33.77)	455 (45.50)
B	*Cys—Lys—Asp*	117 (21.83)	174 (17.40)
C	*Arg—Lys—Gly*	49 (9.14)	157 (15.70)
D	*Cys—Lys—Gly*	5 (0.93)	4 (0.40)
E	*Arg—Gln—Asp*	154(28.73)	176(17.60)
F	*Arg—Gln—Gly*	24(4.48)	29 (2.90)
G	*Cys—Gln—Gly*	1 (0.19)	4 (0.40)
H	*Cys—Gln—Asp*	5 (0.93)	1 (0.10)

**Table 6:** Association of haplotypes with polymorphisms of LMP2, LMP7 and TAP1-2 with risk of ESCC in the Kazakh population.

Haplotypes^b^	Cases n (%)	Controls n (%)	*P*^a^	OR (95% CI)^a^
*A* = *Arg—Lys—Asp*				
–/–	118 (43.90)	149 (29.70)	—	1.00 (reference)
–/A	120 (44.68)	248 (49.60)	0.004**	0.61 (0.44–0.85)
A/A	30 (11.42)	103 (20.70)	<0.001**	0.37 (0.23–0.59)
B = *Cys—Lys—Asp*				
–/–	164(61.19)	341(68.17)	—	1.00 (reference)
–/B	91 (34.06)	144 (28.80)	0.10	1.31 (0.95–1.81)
B/B	13 (4.75)	15(3.03)	0.15	1.80 (0.84–3.88)
C = *Arg—Lys—Gly*				
–/–	222 (82.65)	356 (71.08)	—	1.00 (reference)
–/C	44 (16.52)	132 (26.46)	0.001**	0.54 (0.37–0.78)
C/C	2(0.83)	12 (2.46)	0.09	0.27 (0.06–1.21)
D *= Cys—Lys—Gly*				
–/–	263 (98.21)	496 (99.22)	—	1.00 (reference)
–/D	5 (1.79)	4 (0.78)	0.29	2.36 (0.638.85)
D/D	0 (0.00)	0 (0.00)	1.00	—
E = Arg—Gln—Asp				
–/–	136 (50.88)	340 (67.90)	—	1.00 (reference)
–/E	110 (40.88)	145 (29.00)	<0.001**	1.90 (1.38–2.61)
E/E	22 (8.24)	15(3.10)	<0.001**	3.67 (1.85–7.28)
F = *Arg—Gln—Gly*				
–/–	244 (91.22)	471 (94.13)	—	1.00 (reference)
–/F	23 (8.58)	29 (5.79)	0.17	1.53 (0.87–2.70)
F/F	1 (0.20)	0 (0.00)	1.00	—
G = *Cys—Gln—Gly*				
–/–	267 (99.59)	496 (99.22)	—	1.00 (reference)
–/G	1 (0.41)	4 (0.78)	0.66	0.46 (0.05–4.18)
G/G	0 (0.00)	0 (0.00)	1.00	—
H *= Cys—Gln—Asp*				
–/–	263 (98.21)	499 (99.71)	—	1.00 (reference)
–/H	5 (1.78)	1 (0.29)	0.02*	1.02(1.00–1.03)
H/H	0 (0.00)	0 (0.00)	1.00	—

a Logistic regression model, adjusted by gender, age, smoking and drinking.

b Symbol (–) denotes any haplotype. For example,–/A indicates the A haplotype in combination with any other haplotypes.

“*”represents *p*<0.05,

“**”indicates *p*<0.01

### Relationship between HPV infection in Kazakh patients with ESCC and LMP2/LMP7 genotypes

The results of genotype frequency analysis for LMP2/LMP7 gene polymorphisms in HPV-positive and HPV-negative patients are summarized in Table 3 and Table 4. There was no association between LMP2/LMP7 and HPV infection in Kazakh patients with ESCC (*p*>0.05).

## Discussion

ESCC is a polygenic hereditary disease that can be affected by genetic factors [27–30]. In our study, the association between LMP2 and LMP7 polymorphisms and the risk of ESCC in the Kazakh population was investigated.

According to our results, heterozygous LMP2 R/C and homozygous LMP2 C/C genotypes are risk factors for ESCC in the Kazakh population. This finding differed from that of Xiang Ma[24] and Bangwei Cao’s [22], which revealed no statistical correlation between LMP2–60 polymorphism and ovarian and gastric cancers.

However, one study did find that LMP2 plays a role in the growth of multiple myeloma and acute myeloid leukemia but that LMP7 is not associated with these hematological malignancies [31]. Nevertheless, in our study, the heterozygous LMP7 Q/K genotype was associated with significantly decreased risk of ESCC in the Kazakh population. This finding was also not consistent with previous results showing that LMP7–145 increased susceptibility to gastric cancer, ovarian cancer and ESCC in a Chinese Han population [11, 22, 24]. The discrepancy between these results and our findings may be attributed to the fact that the same polymorphism may have diverse genetic effects on different types of cancer, ethnicities and races [32, 33]. When considered together, Kazakh patients possessing both the LMP2 R/C+C/C genotype and the wild-type homozygous LMP7 Q/Q genotype exhibited a higher risk of developing ESCC, further illustrating the existence of linkage disequilibrium in LMP polymorphisms.

In subgroup analysis, the LMP2 R/C genotype was associated with increased tumor invasion depth in ESCC, suggesting that genetic variation in immune response genes may play a role in ESCC progression. Additionally, no association between LMP7–145 polymorphism genotypes and ESCC clinicopathological characteristics of the Kazakh population was observed in our study. Song [11] revealed that LMP7 polymorphism increases the risk of lymph node and tumor distant metastasis in ovarian cancer.

The LMP2–60 C/C and R/C+C/C genotypes were associated with an increased risk of ESCC in Kazakh patients aged ≥57 years but not in those aged <57 years. This indicates that carcinogenesis is an accumulation of genetic events during aging [34]. Additionally, the increased risk of ESCC in the elderly also demonstrates that gene–environment interactions may be involved in carcinogenesis. A significantly elevated risk was identified in male subjects, consistent with the results of some studies suggesting that female sex hormone exposure may play a protective role in the development of this cancer. Nonetheless, the LMP7–145 Q/K and QK+KK genotypes were associated with a decreased risk of ESCC in male patients aged ≥57 years but not in male patients aged <57 years.

Our previous studies found that TAP1 polymorphisms could increase susceptibility to ESCC in the Kazakh population [35]. Moreover, TAP1, LMP2 and LMP7 are all located within a narrow region of the class II cluster of the major histocompatibility complex on chromosome 6 [14]. In our study, eight haplotypes were constructed to analyze linkage disequilibrium for LMP2, LMP7 and TAP1, and our results indicated that haplotypes A and E were more common in both the patients and control individuals. Haplotype A, including wild-type homozygous LMP2/TAP1 and mutant LMP7, decreased the risk of ESCC in the Kazakh population. Moreover, haplotype E, including wild-type homozygous LMP2/LMP7/TAP1, increased the risk of ESCC in this population. These results indicate that the LMP7–145 (Gln–Lys) gene variation may weaken the susceptibility of the haplotype to ESCC in the Kazakh population. However, further studies are needed to clarify this association.

To date, many studies have confirmed that HPV is present in ESCC samples, with infection rates ranging from 17.1 [36] to 78.11% [22]. One study reported an infection rate of 100%, which was confirmed by detection of the HPV16 E6 and E7 genes in a high-risk area in Anyang, Henan [37]. Some reports even found that HPV infection is an important factor contributing to the high incidence of ESCC in the Kazakh population in Xinjiang [35]. Indeed, a study on cervical cancer showed that the genetic makeup of host immune response genes may be responsible for biological variability to HPV infection [38]. Our study sought to assess a connection between LMP2/LMP7 polymorphism and HPV infection, though we found no association between these variables. Our results indicate that HPV infection and LMP2/LMP7 polymorphisms may function independently to influence the risk of ESCC in the Kazakh population, a conclusion that supports the research of Cao [22] in Anyang, China.

In conclusion, to the best of our knowledge, the present study is the first to demonstrate that LMP2/LMP7 polymorphisms are associated with the risk of ESCC in the Kazakh population, a Chinese ethnic minority. LMP2 can be considered a risk factor for ESCC and LMP7 as a protective factor against ESCC in the Kazakh population. Additional studies with larger sample sizes and the inclusion of various populations, as well as functional studies, are required to verify the present preliminary findings.

## Supporting information

S1 Fig LMP2 gene enzyme digestion and sequencing map. (PDF)

S2 Fig LMP7 gene enzyme digestion and sequencing map. (PDF)

S3 Fig Bioinformatics tools to analyze LMP2 gene expression. (PDF)

S1 Table Primer sets used for amplification and sequencing of LMP/TAP genes. (PDF)

S2 Table Hardy-Weinberg test for cases and controls. (PDF)

S3 Table Epidemiological questionnaire in Chinese and in English. (PDF)
