# Supplementary material for: Genetic variability in LMP2 and LMP7 is associated with the risk of esophageal squamous cell carcinoma in the Kazakh population but is not associated with HPV infection
Source: PLoS One. 2017 Oct 26;12(10):e0186319. doi: 10.1371/journal.pone.0186319 (PMC5657974; doi:10.1371/journal.pone.0186319)
Supplement: S1 Fig — (PDF) [file pone.0186319.s001.pdf]

Supplement figure 1

A

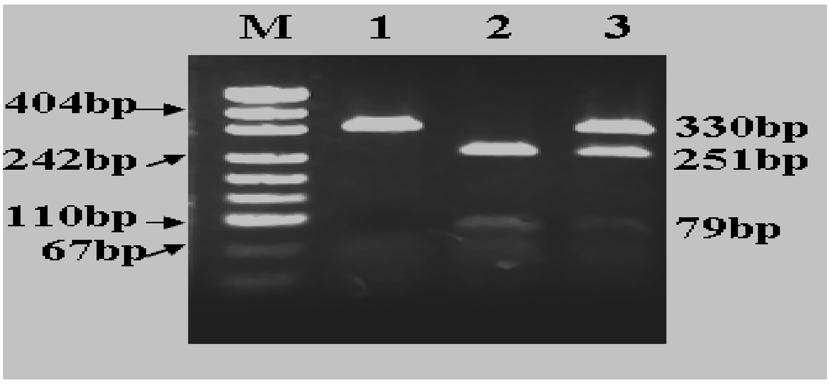

B

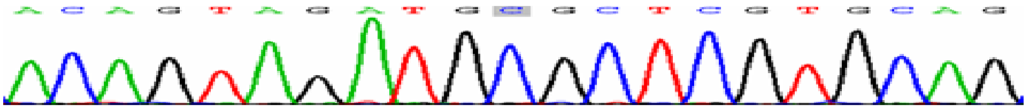

Sequencing map of the genotype for the LMP2 R/R

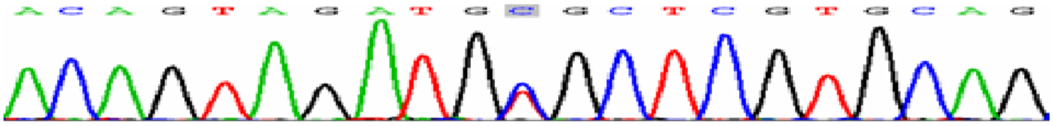

Sequencing map of the genotype for the LMP2 R/C

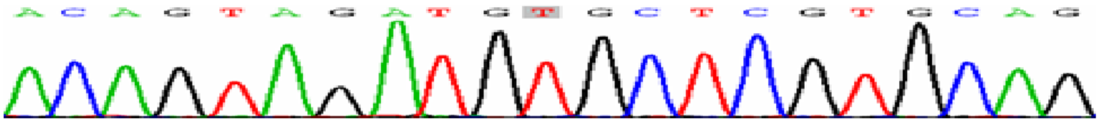

Sequencing map of the genotype for the LMP2 C/C

Fig.1A. LMP2 gene enzyme digestion and sequencing map

M : DNA marker; 1:LMP2C/C ;2:LMP2 R/R ;3:LMP2 R/C

1B. Sequencing map of the genotype for the LMP2 genetypes
