# Supplementary material for: Genetic variability in LMP2 and LMP7 is associated with the risk of esophageal squamous cell carcinoma in the Kazakh population but is not associated with HPV infection
Source: PLoS One. 2017 Oct 26;12(10):e0186319. doi: 10.1371/journal.pone.0186319 (PMC5657974; doi:10.1371/journal.pone.0186319)
Supplement: S2 Fig — (PDF) [file pone.0186319.s002.pdf]

Supplement figure 2

A

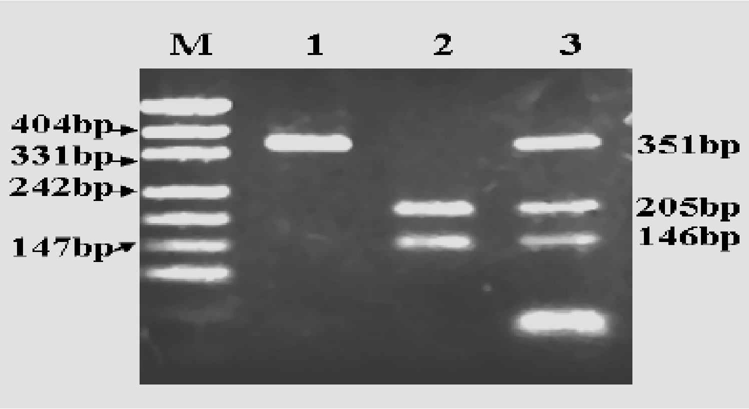

B

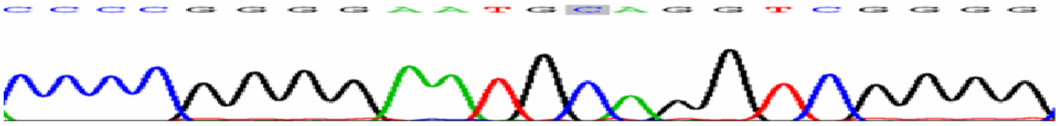

Sequencing map of the genotype for the LMP7 Q/Q

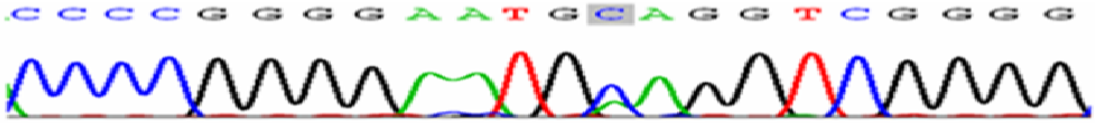

Sequencing map of the genotype for the LMP7 Q/K

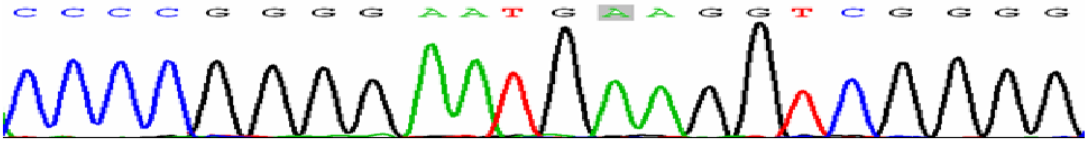

Sequencing map of the genotype for the LMP7 K/K

Fig.2A. LMP7 gene enzyme digestion and sequencing map

M : DNA marker; 1:LMP7K/K ;2:LMP7Q/Q ;3:LMP7Q/K

2B. Sequencing map of the genotype for the LMP7 genotypes
