# Supplementary figures and images for: Genetic variability in LMP2 and LMP7 is associated with the risk of esophageal squamous cell carcinoma in the Kazakh population but is not associated with HPV infection
Source: PLoS One. 2017 Oct 26;12(10):e0186319. doi: 10.1371/journal.pone.0186319 (PMC5657974; doi:10.1371/journal.pone.0186319)

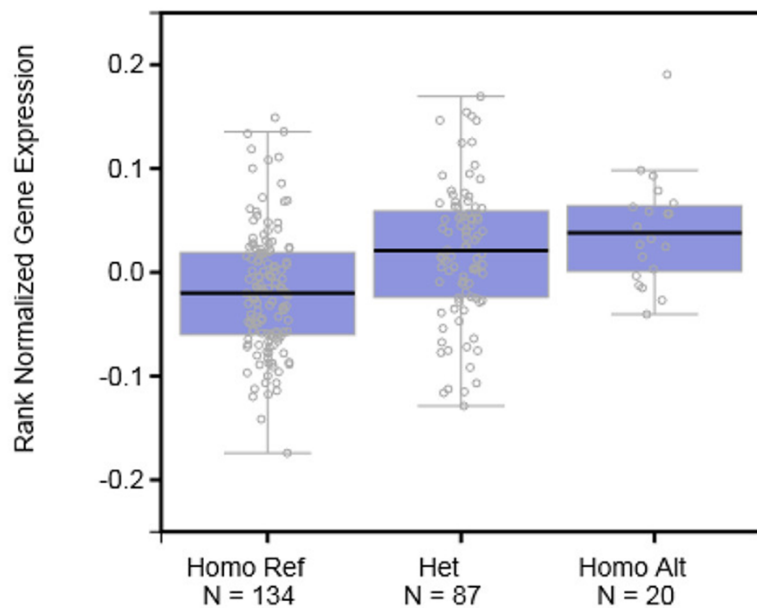

Figure3 Bioinformatics tools to analyze LMP2/LMP7 gene expression .

Supplement: S3 Fig — (PDF) [file pone.0186319.s003.pdf]
