# Supplementary material for: Genetic variability in LMP2 and LMP7 is associated with the risk of esophageal squamous cell carcinoma in the Kazakh population but is not associated with HPV infection
Source: PLoS One. 2017 Oct 26;12(10):e0186319. doi: 10.1371/journal.pone.0186319 (PMC5657974; doi:10.1371/journal.pone.0186319)
Supplement: S1 Table — (PDF) [file pone.0186319.s004.pdf]

S1 Table Primer sets used for amplification and sequencing of LMP/TAP genes

Gene	Amino acid position	Substitution for the nucleotide	PCR primers (sense/antisense)	Length (bp)	Substitution for the amino acid
LMP2	60	CGC→TGC	5′-CTCCACTTTACAGATGCAGA-3′<br>5′-ACTTGGTGACTGTTGACTCC-3′	331	Arg→Cys
LMP7	145	CAG→AAG	5′-TCATGGCGCTACTAGATGTATG-3′<br>5′-AACTCTTTGTCCTAACTTGCAC-3′	351	Gln→Lys
