# Supplementary material for: Genetic variability in LMP2 and LMP7 is associated with the risk of esophageal squamous cell carcinoma in the Kazakh population but is not associated with HPV infection
Source: PLoS One. 2017 Oct 26;12(10):e0186319. doi: 10.1371/journal.pone.0186319 (PMC5657974; doi:10.1371/journal.pone.0186319)
Supplement: S2 Table — (PDF) [file pone.0186319.s005.pdf]

TableA Cases of Hardy-Weinberg Test

Gene	Genotype	Observations	Predictive value	X^2	P#
LMP2	R/R	111	115.7		
	R/C	130	120.8	0.855	0.652
	C/C	27	31.5		
LMP7	Q/Q	218	211.3		
	Q/K	40	53.3	5.699	0.058
	K/K	10	3.4		

#P>0.05

TableB Controls of Hardy-Weinberg Test

Gene	Genotype	Observations	Predictive value	X^2	P#
LMP2	R/R	261	266.5		
	R/C	208	197.1	0.895	0.639
	C/C	31	36.5		
LMP7	Q/Q	342	346.9		
	Q/K	149	139.1	1.470	0.479
	K/K	9	13.9		

#P>0.05
