# Supplementary material for: Genetic variability in LMP2 and LMP7 is associated with the risk of esophageal squamous cell carcinoma in the Kazakh population but is not associated with HPV infection
Source: PLoS One. 2017 Oct 26;12(10):e0186319. doi: 10.1371/journal.pone.0186319 (PMC5657974; doi:10.1371/journal.pone.0186319)
Supplement: S3 Table — (PDF) [file pone.0186319.s006.pdf]

姓名________________　　　　　　　　　　ID#:|　|　|　|　|　|　|　|　|　|　|　|　|

"食管癌早诊早治项目"内镜检查、治疗知情同意书

食管癌是这一地区的常见病，很多当地居民死于食管癌。原因就是治疗太晚，这次检查就是要早期发现癌症及时治疗，取得长期生存的好效果。同时还可以通过筛查早期发现、早期诊断和早期治疗食管癌，总结经验向全国推广，尽快降低食管癌死亡率，基本控制高危地区食管癌危害，造福人民。

<u>检查和治疗过程</u>

我们邀请年龄在40—69岁的当地的居民，参加内镜检查，在内镜检查时除肉眼观察，还要从病变处取几块组织用以准确诊断。如果发现患有食管癌或癌前病变（如重度不典型增生等）将按每人不同病况，安排不同的治疗。患重度不典型增生和早期癌症者可行内镜下粘膜切除手术。中晚期患者可行手术或放疗。

<u>参加检查和治疗的危险性</u>

内镜检查和治疗时一般情况下都很安全。但也有个别情况，检查和治疗时发生出血、食管穿孔和药物反应（如碘过敏）等。对这些情况医生会很好预防，即使发生也会及时处理。但是也有可能情况严重，甚至出现生命危险。如果您想进一步了解情况，请与检查小组的医生联系。

<u>参加检查的好处</u>

如果您参加检查，可全面了解你的食管、胃和十二指肠球部的情况，发现肿瘤可及时治疗。

<u>保密原则</u>

您的检查结果，依照相关法律会被严密保存，不被公开。我们将储存本项目的所取的活检标本，并可能在以后的研究中使用，活检标本上贴有带编码的标签，不会出现您的姓名，您的所有信息将会保密。

<u>自愿原则</u>

检查是完全自愿的，是否参加请您自己决定。您可以拒绝参加，您有权利随时退出检查。如果您有任何问题，可与检查小组中人员联系。电话：______________________________；地址：____________________________。

<u>自我声明</u>

我已阅读本知情同意书，理解了全部情况。一些问题已同检查小组人员讨论，并得到满意解决，我同意参加此次内镜检查和治疗。

参加人员签字______________________　　　　　　日期____年____月____日

<u>证人声明</u>

我已经向受检对象宣读和解释了这份知情同意书。她已经理解并同意参加本项目。

证人签字：_________________________　　　　　　日期____年____月____日

“食管癌早诊早治项目”基线调查

——食管癌危险因素调查表

姓名：________________　　调查对象顺序号：|　|　|　|　|　|　|　|　|　|　|　|

性别：（男—1，女—2）|__|　　出生日期：|__|__|__|__|年|__|__|月|__|__|日

身份证号：|__|__|__|__|__|__|__|__|__|__|__|__|__|__|__|　　调查员姓名：________________

调查日期：|__|__|__|__|年|__|__|月|__|__|日

1. 一般情况：

1.1 婚姻状况：|__|
（0=未婚，1=已婚，2=离异，3=丧偶）

1.2 你最高受过何等教育：|__|
（0=未正式上过学，1=小学，2=中学，3=大专及以上）

1.3 你家现在有几口人，平均年收入大约为多少元？
|__|__|人　|__|__|__|__|__|元/年

2. 饮水情况：

2.1 你的饮水主要来源于：|__|
（1=地窖水、池溏水、浅井水 2=湖水，河水
3=深井水、泉水　4=自来水（经处理））

3. 吸烟情况：（0=否，1=是）

类型	是/否	每年/月吸多少？	共吸了多少年？
3.1 纸烟	|__|	|__|__|支/天	|__|__|年
3.2 烟叶	|__|	|__|__|两/月	|__|__|年

4. 饮酒情况：（0=否，1=是）

类型	是/否	每天喝多少？	共喝多少年
4.1 啤酒	|__|	|__|__|__|__|毫升/天	|__|__|年
4.2 白酒	|__|	|__|__|两/天	|__|__|年
4.3 果酒	|__|	|__|__|两/天	|__|__|年
4.4 其它酒	|__|	|__|__|两/天	|__|__|年

5. 饮茶情况：（0=否，1=是）

类型	是/否	每月喝多少？	共喝了多少年？
5.1 花茶	|__|	|__|两/月	|__|__|年
5.2 绿茶	|__|	|__|两/月	|__|__|年
5.3 红茶	|__|	|__|两/月	|__|__|年

6.饮食习惯：（过去一年内，你是否食用以下食品，请在相应的栏内填入数字）

食品名称	次/周	次/月	次/年	持续数月
6.1 新鲜蔬菜	|__|__|	|__|	|__|__|	|__|__|
6.2 新鲜水果	|__|__|	|__|	|__|__|	|__|__|
6.3 肉蛋奶类	|__|__|	|__|	|__|__|	|__|__|
6.4 豆类食品	|__|__|	|__|	|__|__|	|__|__|
6.5 葱、蒜	|__|__|	|__|	|__|__|	|__|__|
6.6 主食(各点决定)	|__|__|	|__|	|__|__|	|__|__|
6.7 干果干菜	|__|__|	|__|	|__|__|	|__|__|
6.8 腌晒食品	|__|__|	|__|	|__|__|	|__|__|
6.9 油炸食品	|__|__|	|__|	|__|__|	|__|__|
6.10 烫热食品	|__|__|	|__|	|__|__|	|__|__|
6.11 霉变食品	|__|__|	|__|	|__|__|	|__|__|

7.消化系统疾病史：（0=否，1=是）

疾病名称	是/否	诊断时年龄
7.1 胃肠炎	|__|	|__|__|岁
7.2 胃、十二指肠溃疡	|__|	|__|__|岁
7.3 食道炎	|__|	|__|__|岁
7.4 肝炎	|__|	|__|__|岁
7.5 其它(请注明)：(　　　　)		|__|__|岁

8.家族史：（你家中有没有人得过肿瘤，0=否，1=是）

亲属关系	肿瘤名称	人数

9.体格检查结果

身高 cm	体重 kg	脉搏(次/分)	血压 mmhg
|__|__|__|	|__|__|__|	|__|__|__|	|__|__|__|/|__|__|__|

Full name:__________________ ID:_______________________

"Early diagnosis and early treatment of esophageal cancer" informed consent for endoscopy and treatment

Esophageal cancer is a common disease in this region, and many local people died of esophageal cancer. The reason is that the treatment is too late, this examination is to early detection of cancer, timely treatment, and long-term survival of good results. At the same time, the screening of early discovery, early diagnosis and early treatment of esophageal carcinoma and summarize the experience will promote the country as soon as possible to reduce the mortality of esophageal cancer.The basic control of esophageal cancer in high-risk areas of harm, can benefit to the people.

Examination and treatment process

We invited local residents aged 40-69 years of age to undergo endoscopic examination. In endoscopic examination, several tissues are removed from the lesion for accurate diagnosis, in addition to the naked eye. If it is founded to be esophageal cancer or precancerous lesions (such as severe atypical hyperplasia), according to each different condition, we adopted different treatment. Patients with severe dysplasia and early

cancer may undergo endoscopic mucosal resection. Patients with advanced diseases may have surgery or radiotherapy.

Participate in the risk of examination and treatment

Endoscopy and treatment are generally safe. However, there are individual cases of bleeding, perforation of esophagus and drug reactions (such as iodine allergy) during examination and treatment. In these cases, the doctor will be very good prevention, even if it happens will be dealt with in time. But it could be serious, even life-threatening. If you would like to know more about the situation, please contact the doctor of the examination team.

Benefits of participating in the examination

If you participate in the examination, you can fully understand the situation of your esophagus, stomach and duodenal bulb, and found that the tumor can be treated in time.

Confidentiality principle

Your examination results will be closely kept in accordance with the relevant laws and will not be disclosed. We will store the biopsy specimens obtained in this project and may be used in future studies. Labeled labels will not appear on your biopsy specimen and your name will not appear. All your information will be kept confidential.

Voluntary principle

Please make your own decision whether to take part in the examination.

You may refuse to attend, and you have the right to withdraw from the inspection at any time. If you have any questions, contact the inspection team. Telephone: ____________Address__________________

Self declaration

I have read this informed consent to understand all the circumstances. A number of questions have been discussed and satisfactorily resolved by the panel members. I agree to participate in the endoscopic examination and treatment.

Signature of participant_______________ Date:_______________

Witness statement

I have read and explained the informed consent to the receiver. She has understood and agreed to take part in the project.

Signature of witness: _____________ Date:________________

Baseline investigation of “early detection and early treatment of esophageal cancer”

--- esophageal cancer risk factors questionnaire

Name:__________ Object sequence number:________________

Sex (male-1 , female-2) Date of birth:_________________________

ID number:___________ Investigator name______________________

Date of investigation________________

1. General conditions:

1.1 marital status:
0=unmarried,1=married,2=divorced,3=widowed

1.2What is your highest education?
0=No,1=Primary,2=secondary,3=University

1.3 How many people are there in your family now? What is the average annual income?
_______people ________Yuan/year

2. Drinking water condition:

2.1 Your drinking water comes mainly from:
1=cellar water, pond water, Asakai Mizu, 2=lake water, river water3=deep well water, spring water, 4.=treated tap water

3.smoking(0=no,1=yes)

type	Yes/no	How much per day?	How many year?
Cigarettes			
Tobacco			

4. Drinking situation: (0=no,1=yes)

type	Yes/no	How much per day?	How many year?
Beer			
Liquor			
fruit wine			

6.Eating habits:

Food name	Per week	Per month	Per year	months
vegetable				
fresh fruit				
Meat or milk				
beans				
onions, garlic				
staple food				
dried fruits				
salted food				
fried food				
hot food				
moldy food				

7. History of digestive system diseases: (0=no,1=yes)

Name of the disease	Yes/no	age of diagnosis
gastroenteritis		
stomach and duodenal ulcer		
esophagitis		
hepatitis		
other		

8.Family history: (0=no,1=yes)

kinship	name of cancer	People

Other wine							
Tea drinking: (0=no,1=yes)							
Type	Yes/no	How much per day?	How many year?	9.Physical examination results:			
scented tea				Height cm	Weight kg	Pulse/ minute	blood pressure
Green Tea							
Black tea							
